# Autoantibodies and Rheumatologic Manifestations in Hepatitis C Virus Infection

**DOI:** 10.3390/biology10111071

**Published:** 2021-10-20

**Authors:** Marta Priora, Richard Borrelli, Simone Parisi, Maria Chiara Ditto, Cristina Realmuto, Angela Laganà, Chiara Centanaro Di Vittorio, Rosanna Degiovanni, Clara Lisa Peroni, Enrico Fusaro

**Affiliations:** 1 Department of General and Specialistic Medicine, Rheumatology Clinic, Hospital of Mondovì, 12084 Cuneo, Italy; 2 Rheumatology Unit, Department of General and Specialistic Medicine, Azienda Ospedaliero Universitaria Città Della Salute e Della Scienza di Torino, 10126 Turin, Italy; richard.borrelli.posta@gmail.com (R.B.); simone.parisi@hotmail.it (S.P.); mditto@cittadellasalute.to.it (M.C.D.); cristina.realmuto@gmail.com (C.R.); laganaangela50@gmail.com (A.L.); ccentanarodivittorio@cittadellasalute.to.it (C.C.D.V.); rdegiovanni@cittadellasalute.to.it (R.D.); cperoni@cittadellasalute.to.it (C.L.P.); fusaro.reumatorino@gmail.com (E.F.)

**Keywords:** auto-antibodies, hepatitis C virus, rheumatological manifestation

## Abstract

**Simple Summary:**

In patients with HCV, rheumatic manifestations are mostly mediated by immunological mechanisms, rather than being related to the viral infection of extrahepatic tissues. Molecular mimicry of viral antigens, chronic stimulation of B cells and a bystander effect are some of the mechanisms for the development of autoimmune phenomena and lymphoproliferative disorders; these conditions can either be clinical, merely serological or both. Among these patients, the occurrence of auto-antibodies is a finding strictly associated with a chronic infectious trigger since HCV has been proved to induce a B-mediated response shortly after the activation of the innate immune system. Given this scenario, a rheumatic disorder can be found as it might coexist with the HCV infection thus giving an overlap syndrome in some patients. Nevertheless, direct-acting antiviral therapies have largely demonstrated to reduce the damage stemming from both systemic inflammatory phenomena and a persistent immune activation by promoting an early viral eradication.

**Abstract:**

HCV is a virus that can cause chronic infection which can result in a systemic disease that may include many rheumatologic manifestations such as arthritis, myalgia, sicca syndrome, cryoglobulinemia vasculitis as well as other non-rheumatological disorders (renal failure, onco-haematological malignancies). In this population, the high frequency of rheumatoid factor (45–70%), antinuclear (10–40%) and anticardiolipin (15–20%) antibodies is a B-cell mediated finding sustained by the infection. However, the possibility that a primitive rheumatic pathology may coexist with the HCV infection is not to be excluded thus complicating a differential diagnosis between primitive and HCV-related disorders.

## 1. Introduction

Hepatitis C virus (HCV) infection is a health problem of global relevance; its scenario has rapidly changed throughout the years owing to the development of direct-acting antivirals (DAAs). Hepatitis C virus (HCV) is a positive strand RNA virus forming the genus Hepacivirus in the Flaviviridae family. It was identified by Choo et al. in 1989 using the approach of molecular cloning, which was both new and more powerful when compared to the classic virus purification. HCV can enter into hepatocytes by using a combination of various proteins: Cluster of Differentiation 81 (also known as CD81 or tetraspanin, which is expressed on both liver cells and B-lymphocytes), the scavenger receptor class B type I claudins and occludins; such elements confer organ- and species- specificity to the pathogen.

Once inside the liver cell, it can remain in its cytoplasm permanently and cause serious chronic diseases. Its clearance is performed by the host immune response, and it is associated with concomitant inflammatory liver cell injury [1]. Given the expression of CD81 on B-lymphocytes, besides its hepatocellular tropism, HCV also showed a lymphotropic role; in fact, it has been also observed to interact with APC (antigen-presenting cells) such as macrophages and peripheral dendritic cells as well as with monocytes [2].

The recognition of the pathogen is initiated by TLR3 (Toll-like receptor 3) and RIG-I (retinoic acid–inducible gene I); while TLR3 senses dsRNA in endosomes, RIG-I is able to recognise the polyuridine motif of the HCV inside the cell. Once TLR3 is activated, it can activate TRIF, which is a molecule whose ability is to promote IFN-β (interferon- beta); on the other hand, RIG-I recruits mitochondrial antiviral proteins (such as MAVS) as well as the adapter molecule IFN-β promoter stimulator protein 1. These processes allow IRF3 (interferon release factor 3, a transcription factor) to translocate into the nucleus therefore providing for an improvement of the synthesis of IFN-β [3,4,5,6].

The persistent viral stimulation allows for a polyclonal expansion of B-cells [7,8] to be performed therefore causing the appearance of immune-complexes [9] which can cause a wide spectrum of autoimmune and lymphoproliferative disorders; these conditions can either be clinical, merely serological or both [10,11].

Currently available therapies are aimed at reducing the damage stemming from systemic inflammatory phenomena and persistent immune activation associated with continuous viral replication; as observed in both literature and real-life data, once the treatment is administered, the virus can be eradicated within 6 to 24 weeks [2]. Besides the understandable specific benefits for the liver, there is a wide range of extra-hepatic advantages that come from its eradication as the association between HCV and autoimmune disorders is well-known: molecular mimicry of viral antigens, chronic stimulation of B cells and the bystander effect are some of the mechanisms for the development of such autoimmune phenomena [12,13].

## 2. Rheumatologic Manifestations

Extra-hepatic manifestations of HCV infection are clinically present in forty to seventy percent of the patients [14,15,16,17], while rheumatologic manifestations have an incidence of 38% [18,19,20,21,22,23] (Table 1).

These are mostly mediated by immunological mechanisms, rather than being related to the infection of extra-hepatic tissues [24].

### 2.1. Musculoskeletal Involvement

Polyarthralgia and arthritis are the most common rheumatic findings associated with HCV since they have been observed in 40 to 80% of patients, according to several studies on varying populations [25,26].

The articular involvement is usually bilateral, symmetrical, non-deforming and it normally targets small joints, such as the metacarpophalangeal joints, the proximal interphalangeal joint and the wrist, thus mimicking the early stage of rheumatoid arthritis [27,28].

A differential diagnosis is fundamental to discriminate between a primary rheumatic disorder and extra-hepatic complications of HCV replication.

The therapeutic approach depends on the clinical manifestations as it ranges from analgesic drugs for arthralgia to immunosuppressants, NSAIDs (non-steroidal anti-inflammatory drugs) and glucocorticoids for arthritis [29,30].

Even if, to date, the exact correlation between HCV and arthritis is not well-established, various reports have established that the eradication of HCV with the DAAs helped solving articular manifestations [31,32]; these correlations might be explained by examining the molecular mechanisms between HCV and B-lymphocytes. First of all, HCV-envelope E2 protein is a viral protein which is able to bind to CD81 (widely expressed on cell membrane of both hepatocytes and B-cells); this complex can create a bridge with CD21 on the surface of the B-lymphocyte thus reducing the threshold for its activation. Not only does this element binds to the aforementioned clusters of differentiation, but it has also been proved to amplify the VDJ rearrangement frequency; this is thought to be the reason for which patients with HCV infection have an increased risk of t(14;18) translocation. Once this genetic alteration is present, the proto-oncogene Bcl-2 becomes overexpressed; consequently, the apoptosis in these B-lymphocytes is extremely reduced, while their half-life appears to be increased. This condition improves the systemic inflammation, but it also increases the relative risk of lymphomas. In fact, B-cell non-Hodgkin lymphoma (NHL), diffuse large B-cell lymphoma and follicular lymphoma have been positively associated with a chronic HCV infection [33]. However, as observed by Couronne et al. in 2018 [34], B-cell receptors (BCR) from HCV-associated lymphoma patients are not actually able to bind to HCV antigens; additionally, stereotyped BCR sequences that contribute to a highly biased range in HCV-associated B-NHL have been also detected in other HCV-negative B-cell malignancies such as MALT lymphoma, chronic lymphocytic leukaemia, non-malignant B cells with RF activity and non-malignant marginal zone splenic B cell therefore suggesting that HCV-associated lymphoma might originate from precursors with autoimmune properties rather than B cells specifically aimed at eliminating the virus; in this scenario, the chronic inflammatory states that the continuous stimulus given to B-lymphocytes would act as an enhancer, rather than a promoter [35,36,37,38].

Myalgia is another finding that has been found in up to 15% of patients in various studies. Although the mechanisms of this condition are still difficult to understand, a direct involvement of HCV in the pathogenesis of diffuse muscle pain has been proved since its viral genomic sequences have been found within muscle fibres of patients with myalgia developed after the diagnosis of HCV infection. Despite some studies report the possible association with fibromyalgia (a condition that causes pain all over the body, sleep problems, fatigue and often emotional and mental distress) in a significant percentage of patients with chronic HCV infection, other analysis failed to confirm this association. In order to assess these unclear results, in 2021 Abdel Moatty A. Afifi et al. tried to determine the frequency and the clinical characteristics of fibromyalgia in a cohort of Egyptian patients with chronic HCV; their study also tried to evaluate its relationship to viral load. Their results allowed to assess that, in these patients, fibromyalgia was actually associated with higher clinometric scores (Widespread pain index- WPI, symptom severity -SS, VAS-pain and points of tenderness) when compared to the control group in a statistically significant way; at the same time, the intensity of the condition was not related to the viral load of Hepatitis C Virus [18].

### 2.2. Connective Tissue Disorders

Around 20–30% of patients with hepatitis C show symptoms of dryness especially in mouth and eyes which are reported as sicca syndrome [39,40,41,42,43]; despite such symptoms, in these patients a defined Sjogren’s syndrome (SSj) appears to be uncommon. Primary SSj is a systemic autoimmune disease that causes secretory gland dysfunctions which leads to a severe dryness of the main mucosal surfaces such as mouth, eyes, nose, pharynx, larynx and vagina; it is defined by the presence of a typical salivary gland histology as well as by anti-SSA (anti-Sjögren’s-syndrome-related antigen A autoantibodies, also called anti-Ro) and/or anti-SSB antibodies (also known as anti-La antibodies). Anti-SSA antibodies have been proved to target two proteins referred to as Ro52 and Ro60; Ro52 is a 52 kDa protein whose function is to bind to specific misfolded ribosomal RNAs thus regulating the degradation of defective molecules. As for Ro60, mice lacking its encoding genes do tend to develop autoimmune disorders, in which it is possible to find antibodies directed against ribosomes and chromatin; glomerulonephritis were observed as well, suggesting the role of Ro60 in preventing systemic autoimmune diseases. Anti-SSB antibodies target a factor known as “La” that binds and protects pre-tRNAs (precursors of transfer RNAs) from exonuclease digestion via sequence-specific recognition; antibodies directed against the Ro/La ribonucleoprotein complexes have been correlated with severe dysfunction of the exocrine glands and a higher prevalence of extra glandular manifestations as well as an earlier onset of the symptoms of such disorders [39].

In HCV-infected patients with sicca syndrome, the histological examination of salivary glands leads to findings which differ from those typically seen in SSj: there is no gland damage [40], CD8+ T-cells infiltrates are more frequently detected and mainly localised in the pericapillary zones rather than in the periductal areas, with no destruction of the ducts [40]. In SSj, focal lymphocytic infiltrates are predominantly composed by CD4+ T cells and the percentage of B cells increases in the late and more severe stages of the disease [41]. Even though literature data on HCV replication in the salivary glands are controversial [42], the presence of the virus in both saliva and salivary epithelial cells has been solidly demonstrated. According to the American-European Consensus Criteria, HCV infection represents an exclusion criterion for the diagnosis of SSj as it stands for an important differential diagnosis in patients with xerophthalmia and xerostomia [39].

### 2.3. Vascular Involvement

As stated in the previous section, HCV is capable of inducing an aberrant activation of B-lymphocytes as well as their prolonged survival therefore increasing the production of antibodies. Before the DAAs age, HCV represented the cause of about 80% of cryoglobulinemia vasculitis; nowadays, such therapies have dramatically reduced the incidence of this clinical scenario [35,36,37]. Circulating cryoglobulins are present in the serum of 40–60% of patients with HCV [24]; its occurrence is frequently asymptomatic, and it is labelled as cryoglobulinemia. However, in approximately 5% of the cases (usually in women over 50 years old) [24], the deposition of cryoglobulins in targeted organs causes tissue damages thus leading to cryoglobulinaemic syndrome. This is an immune complex-mediated small vessel systemic vasculitis, which mainly affects skin, joints, peripheral nerves and kidneys; it is characterised by the presence of serum immunoglobulins that precipitate reversibly at temperatures lower than 37 °C and return to soluble state if the temperature is above this level. Since HCV-related cryoglobulinemia usually shows polyclonal cryoglobulins, it includes both type II and III.

The predictive factors that are most solidly associated with mixed cryoglobulinemia are the elderly age, the long duration of the disease, type II mixed cryoglobulins, a higher level of mixed cryoglobulin serum and B-cell clonal expansion [10,33]. The incidence of mortality varies within a range between twenty and eighty percent and depends on the involved organ systems. Renal involvement, intestinal ischemia, pulmonary haemorrhage, high cryocrit levels and type II mixed cryoglobulinemia are, among others, associated with severe prognosis [10]. It has been demonstrated that most manifestations of mixed cryoglobulinemia have a strong correlation with the HCV virological response and provide a great response to the clearance of HCV during the antiviral therapy.

Curing and preventing cryoglobulinaemic vasculitis is the core priority, and for this reason, patients should receive a proper antiviral therapy. Owing to long-standing high profile research, the understanding of the viral lifecycle of HCV is very accurate and majorly facilitates the setting of new therapeutic strategies.

To the best of our knowledge, DAAs have proved effective against a wide spectrum of HCV genotypes as well as in the treatment of mixed cryoglobulinemia, with an excellent safety profile. They are expected to modify both the incidence of vasculitis stemming from a prolonged history of HCV infection and the therapeutic algorithms in the early stages of the disease [29] as they reduce the viral load therefore determining a significant decrease in the production of antibodies. However, their impact on such elements has not been proved to be effective on the pathogenesis of cryoglobulinemia vasculitis nor they have had a proper impact on the development of the immune-mediated injury once the immune disorder is established. In light of this, patients with severe mixed cryoglobulinemia and, most importantly, with renal and polyneuropathic involvement necessitate control of the disease with rituximab, with or without plasmapheresis, before or in the meanwhile the initiation of optimal antiviral therapy [29]. Other immunosuppressant drugs, such as cyclophosphamide, may also be used in more severe or refractory case [33].

Even though it is more commonly associated with hepatitis B, polyarteritis nodosa (PAN) may also occur in up to 12% of patients with hepatitis C [44]. Cacoub et al. in 1999 [45], studied the incidence of vasculitic manifestations in HCV patients [46]; this study assessed that 19.3% of a group of 161 patients suffering from HCV-related vasculitis had a diagnosis of PAN. The most frequent signs and symptoms found in this cohort were purpura (68%), livedo reticularis (20–60%), arthralgia (61%), weight loss (60%), multiplex mononeuritis (70%), myalgias or weakness (58%), altered arteriography (49%), hypertension (37–55%), abdominal pain (30%), raised creatinine (26%), fever (20%), polyneuropathy (16%), proteinuria (16%), haematuria (16%), intestinal bleeding (16%), diarrhoea (13%) and orchitis (0–7%) [46,47].

Other vasculitis, including Henoch-Schonlein purpura and isolated cutaneous necrotising vasculitis are much rarer [47].

Some studies demonstrate a higher prevalence of HCV infection among patients with systemic lupus erythematosus (SLE) when compared to the general population [48]; however, this statement is still controversial as it is not been confirmed by significant literature. Although a possible pathogenic association between HCV and SLE is yet to be demonstrated and cleared, the problem undoubtedly persists when it comes to differential diagnosis: SLE shares some clinical and serological characteristics of various rheumatic manifestations of chronic hepatitis C, such as arthralgia, myalgia, sicca syndrome, antinuclear antibodies and antiphospholipid antibodies positivity.

Recently, a study conducted by Tiosano et al. [49] has investigated the association between systemic sclerosis (SSc) and chronic HBV (Hepatitis B Virus) and HCV by performing a cross-sectional study with the database of Clalit Health Services (the largest healthcare organisation in Israel); since the results showed an association between HBV/HCV and systemic sclerosis, further studies are required to verify whether common or specific immune mechanisms may be involved as mediators of this mechanisms.

## 3. Auto-Antibodies and HCV

The occurrence of auto-antibodies in HCV patients is a frequent cause of requests for rheumatologic counselling in daily clinical practice.

HCV can promote the production of several auto-antibodies, thus complicating the differential diagnosis between primitive and HCV-related rheumatic disorders [50] (Table 2).

As the evidence shows, at least one serum immunological abnormality is present in up to 50% of HCV-infected patients. In the recent years, various antibodies and their relationships with chronic hepatitis C have been subject to intense studying.

The high frequency of RF (rheumatoid factor, 45–70%), ANA (antinuclear antibodies, 10–40%) and anticardiolipin (ACLA IgG-IgM 20–15%) in HCV patients is a finding strictly associated with a chronic infectious trigger; in contrast, this positivity is not linked to manifestations of connective tissue disorders [51].

### 3.1. Rheumatoid Factor

The most often detected antibody is undoubtedly the rheumatoid factor (RF). It is an immunoglobulin of different possible isotypes (mainly IgM) directed against the Fc fraction of another immunoglobulin G (IgG) [52].

In 1940, Waaler described an antibody directed against serum gamma-globulins that promoted the agglutination of sheep red blood cells sensitised by subagglutinating doses of rabbit antibodies [53]; a similar discovery was previously found in patients with liver cirrhosis by Kurt Meyer in 1922. In 1948, Rose described these antibodies in patients with rheumatoid arthritis [54] and in 1952 they were named RFs because of their association with rheumatoid arthritis (RA).

Natural RFs are generally low-affinity, polyreactive IgM antibodies produced by CD5-positive B cells; the coexistence of RF-positive B cells and non-autoimmune IgG antigen in healthy subjects suggests the existence of a tolerance mechanism.

Although the name might allow to think this antibody is merely associated with rheumatoid arthritis, RF can be found in patients with other non-rheumatic and non-hepatic diseases as well as in healthy subjects: in fact, its role is important in classification criteria but not per se as a diagnostic tool. RF has been reported to increase the clearance of immune-complexes while RF-producing B cells have been seen to behave as antigen-presenting cells (APCs) as they support the immune response against non-self antigens; given these elements, the impact of RF production during infections might be protective for the host [55,56].

### 3.2. Antinuclear Antibodies

The occurrence of antinuclear antibodies (ANA) positivity in HCV patients has been extensively studied. ANA are a spectrum of auto-antibodies directed against various nuclear and cytoplasmic components of the cells. As for the rheumatoid factor, they are useful as serological markers for the classification of many rheumatic diseases rather than their diagnosis.

Despite the clinical associations of ANA with rheumatic and non-rheumatic conditions, the relationship between these antibodies and specific disease manifestations is often unknown since their targets are predominantly intracellular and ubiquitously expressed [57].

In the most recent studies, patients with HCV showed a percentage of ANA positivity between 10% and 40%, while the control group only showed an occurrence of 3% to 4%.

Several investigators have wondered whether patients with HCV and ANA have different disease profiles [58,59], while others have concluded that the ANA positivity is an immunological epiphenomenon that has no influence on the response to therapy or histology [60,61,62].

### 3.3. Other Auto-Antibodies

Other auto-antibodies have been reported in patients with HCV; although, their occurrence is less frequent when compared to ANA and rheumatoid factor; anti-smooth muscle antibody (ASMA), anti-mitochondrial antibody (AMA) and anti-liver kidney microsomal antibody (LKM) are among these [1,6,63,64].

Multiple studies have considered and assessed eventual differences in demographic factors (such as sex, age and race or ethnicity) and/or clinical outcomes (such as sustained virological response, stage of fibrosis and the presence of cirrhosis) between HCV patients with and without auto-antibodies; however, as of today, the results are conflicting and non-conclusive [24,59,60,62,64].

Four studies demonstrated a higher prevalence of auto-antibodies positivity in women [65,66,67]. Only two studies demonstrated decreased rates of sustained virological response in auto-antibodies positive patients [68,69] while seven studies found no difference in sustained virological response rates [24,59,62,64,65,66,70]. In 2018, Gilman et al. [1] conducted a study in which patients with auto-antibodies mainly antinuclear antibody, antimitochondrial antibody, anti smooth muscle antibody and/or anti liver kidney microsomal antibody (LKM) were more likely to develop HCV extrahepatic manifestations, reinforcing the concept that auto-antibodies in HCV patients might be a laboratory phenomenon rather than a different phenotypic expression of infection. In this population, auto-antibodies positivity was widely prevalent, and it was more commonly found in women; auto-antibodies were not associated with an increased prevalence of extrahepatic manifestations and had no impact on the natural history of HCV infection.

In fact, Gilman et al. concluded their study postulating that auto-antibodies should not be routinely checked in HCV patients, unless there are other indications for autoimmune signs or symptoms.

The mechanisms of generation of these antibodies are not completely understood; HCV can trigger a B-mediated response shortly after the activation of the innate immune system. In subjects who do not clear the virus, the humoral immunity driven by B lymphocytes produces specific antibodies which constantly (or repeatedly) fail to inactivate the viral production and replication. Therefore, the persistent stimulation of B cells by continuous HCV replication may induce their dysfunction with abnormal production of antibodies, among which, indeed, the auto-antibodies [32].

Alternatively, another perspective is that the presence of auto-antibodies in HCV patients may be dictated by chronic apoptotic destruction of hepatocytes. Viruses, unlike bacteria and fungi, cannot reproduce on their own and need the resources of a host cell as they are obligate intracellular parasites [71,72]. Antiviral immunity is, in fact, directed against the host cell infected with the virus. Tissue damages, either directly by viruses or as a result of immune aggressions against infected cells results in the release of a large number of tissue antigens [12]. This could explain the high recurrence of ANA seen in patients with acute alcoholic hepatitis and acute viral hepatitis [73]. Additionally, it has been previously postulated that molecular mimicry and similarities between HCV antigens and host antigens are partly responsible for the development of ANA and ASMA [1].

It is utterly important to underline the correlation between these phenomena and autoimmune thyroid diseases, in which thyroid autoantibodies (TAAb) such as thyroid peroxidase (TPOAb), thyroglobulin (TGAb) and thyroid stimulating hormone (TSH) receptor antibodies (TRAb) might be observed [24]. The most widespread clinical manifestation of such diseases remains Hashimoto’s thyroiditis, in the form of subclinical hypothyroidism or clinically apparent hypothyroidism. Hypothyroidism symptoms and the presence of TPOAb and/or TGAb can suitably lead to the diagnosis of the disease. Graves-Basedow disease, an autoantibody-mediated autoimmune disease epitomised by thyrotoxicosis, is caused by the direct stimulation of the thyroid epithelial cells by TRAb. Besides the presence of these elements of autoimmunity, it has been observed how TSH levels typically lower and fT4 and free triiodothyronine levels increase. HCV infection is commonly associated with thyroid autoimmunity. Most of TAAb in chronic HCV carriers has been reported over the years, ranging between 4.5% and 25% [18,20,25]. The significant span in this range can be ascribed to the various methodological applications and the features of the analysed populations.

Notably, a concern about the presence of TAABs, preceding oral direct-antiviral agents, has been the risk of development of thyroid disease during IFN-based treatment [23]. As illustrated by the literature, in the case of patients who were positive for TAAb before starting IFN-based treatments, such therapy for hepatitis C can lead to the production of TAAb or to a significant increase in TAAb levels. The development of TAAb as a consequence of IFN-based treatments ranges between 1.9% and 40.0% [18,20,24,25]. The literature highlights in particular the direct toxic effect of IFN on thyroid cells without the participation of immunological factors related to the HCV replication [24]. With direct antiviral agents, such a problem has lost most of its significance and, as studies confirm, represents no longer a priority in the clinical management of HCV.

A study by Antonelli C. et al. [23] aimed to investigate the prevalence and characteristics of thyroid disorders as they observed that the prevalence of the thyroid abnormalities was significantly higher in HCV-CV compared to the other groups therefore stating that a careful thyroid surveillance might be advisable in these individuals.

## 4. Conclusions

The association between HCV and autoimmune disorders is well-known. Molecular mimicry of viral antigens by self-antigens, chronic stimulation of B cells and the bystander effect are some of the mechanisms for the development of autoimmune phenomena in hepatitis C.

Many specific studies in literature have extensively inquired into the relation between autoantibodies and HCV/hepatitis C virus infection across remarkably varying populations while showcasing diverse patterns as results.

In these patients, the presence of autoantibodies can induce important clinical implications among many systems (including, besides the liver, the musculo-skeletal system, connective tissue, vascular structures, onco-haematological disorders and renal involvement). In fact, polyarthralgia, arthritis, myalgia, sicca syndrome, cryoglobulinemia, renal failure and lymphomas (as well as other disorders) can be observed. Therefore, an accurate differential diagnosis between primary rheumatic diseases in HCV patients as opposed to HCV-related manifestations is always required thus allowing physicians to consider an appropriate screening protocol for HCV.

It is worth recalling that the recent advent of oral DDAs has strongly changed the therapeutic approach, as they are associated with a great improvement or even the resolution of a lot of both liver-related and extrahepatic complications, including rheumatic ones. However, more studies are necessary to establish the most likely clinical outcomes of disease based on the serological profile of the patients; in fact, to date, this remains one of the biggest limitations of the study as further evaluations concerning this topic are required.

## Figures and Tables

**Table 1:** Rheumatologic manifestations in HCV infection.

Items	Frequency
Polyarthralgia	40–80%
Arthritis	4–5%
Cryoglobulinemia vasculitis	5–40%
Sicca syndrome	20–30%
Polyarteritis nodosa	12–19%
Systemic Lupus Erythematosus	very rare

**Table 2:** Frequency of autoantibodies in chronic hepatitis C.

Type of Autoantibody	Frequency
Rheumatoid factor	45–70%
Cryoglobulins	40–60%
cANCA	10–64%
Anti-C1q	15–31%
Anti-cardiolipin	15–30%
Antinuclear antibodies	10–40%
Anti-smooth muscle	7–27%
Anti-dsDNA	3–25%
Anti-thyroid	4.5–25%
Anti-mitochondrial	1%

## Data Availability

Not applicable.

## References

[1] Gilman A.J., Le A.K., Zhao C., Hoang J., Yasukawa L.A., Weber S.C., Vierling J.M., Nguyen M.H. (2018). Autoantibodies in chronic hepatitis C virus infection: Impact on clinical outcomes and extrahepatic manifestations. BMJ Open Gastroenterol..

[2] Rosso C., Caviglia G.P., Ciruolo M., Ciancio A., Younes R., Olivero A., Giordanino C., Troshina G., Abate M.L., Rizzetto M. (2019). Clinical outcomes in chronic hepatitis C long-term responders to pre-direct antiviral agents: A single-center retrospective study. Minerva Medica.

[3] Bartenschlager R., Cosset F.-L., Lohmann V. (2010). Hepatitis C virus replication cycle. J. Hepatol..

[4] Zignego A., Giannini C., Monti M., Gragnani L. (2007). Hepatitis C virus lymphotropism: Lessons from a decade of studies. Dig. Liver Dis..

[5] Antonelli A., Ferri C., Galeazzi M., Giannitti C., Manno D., Mieli-Vergani G., Menegatti E., Olivieri I., Puoti M., Palazzi C. (2008). HCV infection: Pathogenesis, clinical manifestations and therapy. Clin. Exp. Rheumatol..

[6] Ferri C. (2015). HCV syndrome: A constellation of organ- and non-organ specific autoimmune disorders, B-cell non-Hodgkin’s lymphoma, and cancer. World J. Hepatol..

[7] Priora M., Realmuto C., Parisi S., Ditto M.C., Borrelli R., Peroni C.L., Laganà A., Fusaro E. (2020). Rheumatologic manifestations of hepatitis C in the era of direct-acting antiviral agents. Minerva Gastroenterol. Dietol..

[8] Caussin-Schwemling C., Schmitt C. (2001). Study of the infection of human blood derived monocyte/macrophages with hepatitis C virus in vitro. J. Med. Virol..

[9] Muramatsu M., Kinoshita K., Fagarasan S., Yamada S., Shinkai Y., Honjo T. (2000). Class Switch Recombination and Hypermutation Require Activation-Induced Cytidine Deaminase (AID), a Potential RNA Editing Enzyme. Cell.

[10] Roccatello D., Sciascia S., Rossi D., Solfietti L., Fenoglio R., Menegatti E., Baldovino S. The Challenge of Treating Hepatitis C Virus-Associated Cryoglobulinemic Vasculitis in the Era of Anti-CD20 Monoclonal Antibodies and Direct Antiviral Agents. On-Cotarget [Internet]. 20 June 2017. http://www.oncotarget.com/fulltext/16986.

[11] Backus L.I., Boothroyd D.B., Phillips B.R., Belperio P., Halloran J., Mole L.A. (2011). A Sustained Virologic Response Reduces Risk of All-Cause Mortality in Patients With Hepatitis C. Clin. Gastroenterol. Hepatol..

[12] Kawamura Y., Ikeda K., Arase Y., Yatsuji H., Sezaki H., Hosaka T., Akuta N., Kobayashi M., Suzuki F., Suzuki Y. (2007). Viral Elimination Reduces Incidence of Malignant Lymphoma in Patients with Hepatitis C. Am. J. Med..

[13] van der Meer A.J., Veldt B.J., Feld J.J., Wedemeyer H., Dufour J.-F., Lammert F., Hofmann W.P., de Knegt R.J., Hansen B.E., Janssen H.L.A. (2012). Association between sustained virological response and all-cause mortality among patients with chronic hepatitis C and advanced hepatic fibrosis. JAMA..

[14] Negro F. (2014). Hepatitis C in 2013: HCV causes systemic disorders that can be cured. Nat. Rev. Gastroenterol. Hepatol..

[15] Smith-Palmer J., Cerri K., Valentine W. (2015). Achieving sustained virologic response in hepatitis C: A systematic review of the clinical, economic and quality of life benefits. BMC Infect. Dis..

[16] Maslennikov R., Ivashkin V., Efremova I., Shirokova E. (2021). Immune disorders and rheumatologic manifestations of viral hepa-titis. World J. Gastroenterol..

[17] Cacoub P., Gragnani L., Comarmond C., Zignego A.L. (2014). Extrahepatic manifestations of chronic hepatitis C virus infection. Dig. Liver Dis..

[18] Afifi A.M.A., Elzul D.W., Ahmed N.A., Sallam R.A. (2022). Fibromyalgia syndrome in chronic hepatitis C virus (HCV) infection patients: A potential association and pathogenic role. Egypt. Rheumatol..

[19] Romano C., Cuomo G., Ferrara R., Del Mastro A., Esposito S., Sellitto A., Adinolfi L.E. (2018). Uncommon immune-mediated extrahepatic manifestations of HCV infection. Expert Rev. Clin. Immunol..

[20] Shahab O., Golabi P., Younossi Z.M. (2018). Chronic kidney disease in patients with chronic hepatitis C virus infection. Minerva Gastroenterol. Dietol..

[21] Caviglia G.P., Rosso C., Fagoonee S., Cisarò F., Andrealli A., Smedile A., Pellicano R. (2015). Endocrine manifestations of chronic HCV infection. Minerva Endocrinol..

[22] Marcucci F., Mele A. (2011). Hepatitis viruses and non-Hodgkin lymphoma: Epidemiology, mechanisms of tumorigenesis, and therapeutic opportunities. Blood.

[23] Antonelli A., Ferri C., Fallahi P., Ferrari S.M., Ghinoi A., Rotondi M., Ferrannini E. (2006). Thyroid Disorders in Chronic Hepatitis C Virus Infection. Thyroid.

[24] Narciso-Schiavon J.L., de Schiavon L.L. (2015). Autoantibodies in chronic hepatitis C: A clinical perspective. World J. Hepatol..

[25] Cacoub P., Renou C., Rosenthal E., Cohen P., Loury I., Loustaud-Ratti V., Yamamoto A.-M., Camproux A.-C., Hausfater P., Musset L. (2000). Extrahepatic Manifestations Associated with Hepatitis C Virus Infection: A Prospective Multicenter Study of 321 Patients. Medicine.

[26] Cacoub P., Comarmond C. (2017). New insights into HCV-related rheumatologic disorders: A review. J. Adv. Res..

[27] Smolen J.S., Aletaha D., Barton A., Burmester G.R., Emery P., Firestein G.S., Kavanaugh A., McInnes I.B., Solomon D.H., Strand V. (2018). Rheumatoid arthritis. Nat. Rev. Dis. Primers..

[28] Ferucci E.D., Choromanski T.L., Varney D.T., Ryan H.S., Townshend-Bulson L.J., McMahon B.J., Wener M.H. (2017). Prevalence and correlates of hepatitis C virus–associated inflammatory arthritis in a population-based cohort. Semin. Arthritis Rheum..

[29] Cacoub P., Commarmond C., Sadoun D., Desbois A.C. (2017). Hepatitis C Virus Infection and Rheumatic Diseases: The Impact of Direct-Acting Antiviral Agents. Rheum Dis. Clin. N. Am..

[30] Palazzi C., Olivieri I., Cacciatore P., Pennese E., D’Amico E. (2005). Difficulties in the differential diagnosis between primitive rheu-matic diseases and hepatitis C virus-related disorders. Clin. Exp. Rheumatol..

[31] Buskila D., Shnaider A., Neumann L., Lorber M., Zilberman D., Hilzenrat N., Kuperman O.J., Sikuler E. (1998). Musculoskeletal manifestations and auto-antibody profile in 90 hepatitis C virus infected Israeli patients. Semin Arthritis Rheum..

[32] Soriano V., Labarga P., Fernandez-Montero J.V., de Mendoza C., Esposito I., Benítez-Gutiérrez L., Barreiro P. (2015). Hepatitis C cure with antiviral therapy—Benefits beyond the liver. Antivir. Ther..

[33] Zignego A.L., Ferri C., Giannini C., La Civita L., Careccia G., Longombardo G., Bellesi G., Caracciolo F., Thiers V., Gentilini P. (1997). Hepatitis C virus infection in mixed cry-oglobulinemia and B-cell non-Hodgkin’s lymphoma: Evidence for a pathogenetic role. Arch Virol..

[34] Couronné L., Bachy E., Roulland S., Nadel B., Davi F., Armand M., Canioni D., Michot J.-M., Visco C., Arcaini L. (2018). From hepatitis C virus infection to B-cell lymphoma. Ann. Oncol..

[35] Fernandes B., Dias E., Mascarenhas-Saraiva M., Bernardes M., Costa L., Cardoso H., Macedo G. (2019). Rheumatologic manifestations of hepatic diseases. Ann. Gastroenterol..

[36] Zignego A., Ferri C., Pileri S., Caini P., Bianchi F. (2007). Italian Association of the Study of Liver Commission on Extrahepatic Manifestations of HCV infection. Extrahepatic manifestations of Hepatitis C Virus infection: A general overview and guidelines for a clinical approach. Dig. Liver Dis..

[37] Shiffman M.L., Benhamou Y. (2015). Cure of HCV related liver disease. Liver Int..

[38] Freni M.A., Artuso D., Gerken G., Spanti C., Marafioti T., Alessi N., Spadaro A., Ajello A., Ferraù O. (1995). Focal lymphocytic aggregates in chronic hepatitis C: Occurrence, immunohistochemical characterization, and relation to markers of autoimmunity. Hepatology.

[39] Shiboski C.H., Shiboski S.C., Seror R., Criswell L.A., Labetoulle M., Lietman T.M., Scofield H., Vitali C., Bowman S.J., Mariette X. (2017). 2016 American College of Rheumatol-ogy/European League Against Rheumatism classification criteria for primary Sjögren’s syndrome: A consensus and da-ta-driven methodology involving three international patient cohorts. Ann. Rheum. Dis..

[40] Vitali C. (2011). Immunopathologic differences of Sjögren’s syndrome versus sicca syndrome in HCV and HIV infection. Arthritis Res. Ther..

[41] Carrozzo M., Scally K. (2014). Oral manifestations of hepatitis C virus infection. World J. Gastroenterol..

[42] Su F.-H., Su C.-T., Chang S.-N., Chen P.-C., Sung F.-C., Lin C.-C., Yeh C.-C. (2012). Association of Hepatitis C Virus Infection With Risk of ESRD: A Population-Based Study. Am. J. Kidney Dis..

[43] Carrozzo M. (2008). Oral diseases associated with hepatitis C virus infection. Part 1. sialadenitis and salivary glands lymphoma. Oral Dis..

[44] Saadoun D., Terrier B., Semoun O., Sene D., Maisonobe T., Musset L., Amoura Z., Resche-Rigon M., Cacoub P. (2010). Hepatitis C virus-associated polyarteritis nodosa. Arthritis Rheum..

[45] Cacoub P., Poynard T., Ghillani P., Charlotte F., Olivi M., Piette J.C., Opolon P., Multivirc Group (1999). Extrahepatic manifestations of chronic hepatitis C. Arthritis Rheum..

[46] Wang C.-R., Tsai H.-W. (2021). Human hepatitis viruses-associated cutaneous and systemic vasculitis. World J. Gastroenterol..

[47] Ramos-Casals M., Muñoz S., Medina F., Jara L.-J., Rosas J., Calvo-Alen J., Brito-Zerón P., Forns X., Sánchez-Tapias J.-M. (2009). Systemic Autoimmune Diseases in Patients with Hepatitis C Virus Infection: Characterization of 1020 Cases (The HISPAMEC Registry). J. Rheumatol..

[48] Ahmed M.M., Berney S.M., Wolf R.E., Hearth-Holmes M., Hayat S., Mubashir E., Vanderheyde H., Chang W., King J.W. (2006). Prevalence of active hepatitis C virus in-fection in patients with systemic lupus erythematosus. Am. J. Med. Sci..

[49] Tiosano S., Cohen A.D., Amital H. (2019). The association between hepatitis B, hepatitis C and systemic sclerosis: A cross-sectional study. Curr. Opin. Rheumatol..

[50] Palazzi C., Buskila D., D’Angelo S., D’Amico E., Olivieri I. (2012). Autoantibodies in patients with chronic hepatitis C virus infection: Pitfalls for the diagnosis of rheumatic diseases. Autoimmun. Rev..

[51] Moll J., Isailovic N., De Santis M., Selmi C. (2019). Rheumatoid Factors in Hepatitis B and C Infections: Connecting Viruses, Autoimmunity, and Cancer. Isr Med. Assoc. J. luglio.

[52] Ingegnoli F., Castelli R., Gualtierotti R. (2013). Rheumatoid Factors: Clinical Applications. Dis. Markers.

[53] Waaler E. (1940). On the occurence of a factor in human serum activating the specific agglutination of sheep blood corpuscles. Acta Pathol. Microbiol. Scand..

[54] Rose H.M. (1948). Differential agglutination of normal and sensitized sheep erythrocytes by sera of patients with rheumatoid ar-thritis. Soc. Exp. Biol. Medicine..

[55] Newkirk M.M. (2002). Rheumatoid Factors: Host Resistance or Autoimmunity?. Clin. Immunol..

[56] Westwood O.M.R., Nelson P.N., Hay F.C. (2006). Rheumatoid factors: What’s new?. Rheumatology.

[57] Litwin C.M., Rourk A.R. (2017). Anti-ENA antibody profiles in patients with hepatitis C virus infection. J. Clin. Lab. Anal..

[58] Chretien P., Chousterman M., Alsamad I.A., Ozenne V., Rosa I., Barrault C., Lons T., Hagege H. (2009). Non-organ-specific autoantibodies in chronic hepatitis C patients: Association with histological activity and fibrosis. J. Autoimmun..

[59] Williams M.J., Lawson A., Neal K.R., Ryder S.D., Irving W.L., Trent HCV Group (2009). Autoantibodies in chronic hepatitis C virus in-fection and their association with disease profile. J. Viral Hepat..

[60] Bai L., Feng Z.-R., Lu H.-Y., Li W.-G., Yu M., Xu X.-Y. (2009). Prevalence of antinuclear and anti-liver-kidney-microsome type-1 antibodies in patients with chronic hepatitis C in China. Chin. Med J..

[61] Acay A., Demir K., Asik G., Tunay H., Acarturk G. (2015). Assessment of the Frequency of Autoantibodies in Chronic Viral Hepati-tis. Pak J. Med. Sci..

[62] Mauss S., Berger F., Schober A., Moog G., Heyne R., John C., Pape S., Hueppe D., Pfeiffer-Vornkahl H., Alshuth U. (2013). Screening for autoantibodies in chronic hepatitis C pa-tients has no effect on treatment initiation or outcome. J. Viral Hepat..

[63] Saadoun D., Sadallah S., Trendelenburg M., Limal N., Sene D., Piette J.C., Schifferli J.A., Cacoub P. (2006). Anti-C1q antibodies in hepatitis C virus infec-tion. Clin. Exp. Immunol..

[64] Chen C.-H., Lee C.-M., Chen C.-H., Hu T.-H., Wang J.-H., Hung C.-H., Chung C., Lu S. (2010). Prevalence and clinical relevance of serum autoanti-bodies in patients with chronic hepatitis C. Chang Gung Med. J..

[65] Cassani F., Cataleta M., Valentini P., Muratori P., Giostra F., Francesconi R., Lenzi M., Bianchi G., Zauli D., Bianchi F.B. (1997). Serum autoantibodies in chronic hepatitis C: Comparison with autoimmune hepatitis and impact on the disease profile. Hepatology.

[66] Yee L.J., Kelleher P., Goldin R., Marshall S., Thomas H.C., Alberti A., Chiaramonte M., Braconier J.-H., Hall A.J., Thursz M.R. (2004). Antinuclear antibodies (ANA) in chronic hepatitis C virus infection: Correlates of positivity and clinical relevance. J. Viral Hepat..

[67] Hsieh M.-Y., Dai C.-Y., Lee L.-P., Huang J.-F., Tsai W.-C., Hou N.-J., Lin Z.-Y., Chen S.-C., Wang L.-Y., Chang W.-Y. (2007). Antinuclear antibody is associated with a more advanced fibrosis and lower RNA levels of hepatitis C virus in patients with chronic hepatitis C. J. Clin. Pathol..

[68] Hsieh M.-Y., Dai C.-Y., Lee L.-P., Huang J.-F., Chuang W.-L., Hou N.-J., Lin Z.-Y., Chen S.-C., Wang L.-Y., Chang W.-Y. (2012). Antinuclear antibody titer and treatment response to peginterferon plus ribavirin for chronic hepatitis C patients. Kaohsiung J. Med. Sci..

[69] Pérez R., Pravia R., Artímez M.L., Linares A., Lombraña J.L., Pérez-López R., Miguel G.S., Pons F., Suárez F., Caro-Patón A. (1997). A comparison between two induction re-gimes for the interferon treatment of chronic hepatitis C. Response related factors. Rev. Esp. Enferm. Dig..

[70] Muratori P., Muratori L., Guidi M., Granito A., Susca M., Lenzi M., Bianchi F.B. (2005). Clinical impact of non-organ-specific autoantibod-ies on the response to combined antiviral treatment in patients with hepatitis C. Clin. Infect. Dis..

[71] Yin J., Redovich J. (2018). Kinetic Modeling of Virus Growth in Cells. Microbiol. Mol. Biol. Rev..

[72] Jara L.J., Medina G., Saavedra M.A. (2018). Autoimmune manifestations of infections. Curr. Opin. Rheumatol..

[73] Lian M., Hua J., Sheng L., Qiu D.K. (2013). Prevalence and significance of autoantibodies in patients with alcoholic liver disease. J. Dig. Dis..

